# Mutations in the prostate specific antigen (*PSA*/*KLK3*) correlate with male infertility

**DOI:** 10.1038/s41598-017-10866-1

**Published:** 2017-09-11

**Authors:** Nishi Gupta, Digumarthi V. S. Sudhakar, Pravin Kumar Gangwar, Satya Narayan Sankhwar, Nalini J. Gupta, Baidyanath Chakraborty, Kumarasamy Thangaraj, Gopal Gupta, Singh Rajender

**Affiliations:** 1 Central Drug Research Institute, Lucknow, India; 2 Centre for Cellular and Molecular Biology, Hyderabad, India; 3 King George’s Medical University, Lucknow, India; 4 Institute of Reproductive Medicine, Kolkata, India

## Abstract

Prostate specific antigen (PSA/KLK3) is known to be the chief executor of the fragmentation of semenogelins, dissolution of semen coagulum, thereby releasing sperm for active motility. Recent research has found that semenogelins also play significant roles in sperm fertility by affecting hyaluronidase activity, capacitation and motility, thereby making PSA important for sperm fertility beyond simple semen liquefaction. PSA level in semen has been shown to correlate with sperm motility, suggesting that PSA level/activity can affect fertility. However, no study investigating the genetic variations in the *KLK3*/*PSA* gene in male fertility has been undertaken. We analyzed the complete coding region of the *KLK3* gene in ethnically matched 875 infertile and 290 fertile men to find if genetic variations in *KLK3* correlate with infertility. Interestingly, this study identified 28 substitutions, of which 8 were novel (not available in public databases). Statistical comparison of the genotype frequencies showed that five SNPs, rs266881 (OR = 2.92, P < 0.0001), rs174776 (OR = 1.91, P < 0.0001), rs266875 (OR = 1.44, P = 0.016), rs35192866 (OR = 4.48, P = 0.025) and rs1810020 (OR = 2.08, P = 0.034) correlated with an increased risk of infertility. On the other hand, c.206 + 235 T > C, was more freuqent in the control group, showing protective association. Our findings suggest that polymorphisms in the *KLK3* gene correlate with infertility risk.

## Introduction

Immediately upon ejaculation, semenogelins (secreted by seminal vesicle) form a coagulum after coming in contact with zinc ions. Semenogelin fibers create a dense network to restrict the motility of spermatozoa; however, it has been suggested that it may involve motility restriction methods beyond simple physical hindrance. Complete arrest of the mobility of sperm flagellum suggests that the semenogelins inhibit spermatozoa motility by associating with a cell surface component localized on the flagellum of each spermatozoon^1–3^. Semen liquefaction occurs within 5–20 minutes of ejaculation. The semenogelins initiate their own degradation by chelating zinc ions as the latter activates a network of kallikrein related peptidases^4^, resulting in the dissolution of semen coagulum and activation of sperm progressive motility^5–7^. Prostate specific antigen (PSA) or kallikrein related peptidase 3 (KLK3) is one of the most abundant proteins in the secretion of normal human prostate epithelium and seminal plasma^8^. PSA is an androgen dependent 30KDa glycoprotein with chymotrypsin like enzymatic activity^9^ and plays a major role in the fragmentation of seminal vesicle secreted proteins (semenogelins). It has been suggested that in addition to facilitating coagulum liquefaction, PSA might activate a motility-activating peptide^10^.

Studies on semen liquefaction have shown that PSA degrades semenogelins preferentially at specific sites^11, 12^. The semenogelins are considered to be the precursor molecules, whose degradation yields a number of polypeptides that have different biological functions, such as increasing sperm hyaluronidase activity^13^, hyper-polarization of sperm plasma membrane^14, 15^, anti-bacterial activity^16^, and prevention of sperm capacitation, O2^.−^ synthesis and hyperactivated motility^2, 10, 15^. Furthermore, they bind and/or interact with a number of proteins such as fibronectin^2, 17^, CD52^18^, protein C inhibitor^19^, heparin^20^, and participate in the formation of a macromolecular complex with clusterin, lactotransferrin and eppin^21^. Semenogelins and their degradation products are also thought to affect sperm fertility by increasing thyrotropin releasing hormone like action, promoting zinc shuttling and inhibin like activities^22^. Therefore, by facilitating semenogelins degradation, PSA serves functions that are important for sperm fertility, apart from releasing motile sperm from semen coagulum.

Optimal pace of semenogelins degradation is critical for fertility as they must fragment for sperm release and their presence is important for inhibiting premature capacitation. As mentioned above, PSA is the chief peptidase behind semenogelins degradation and generation of active peptides. Studies till date viewed PSA from semen liquefaction point of view, looking for correlation between PSA level and sperm motility^23^. Since PSA mediated semenogelin degradation serves functions beyond sperm release, its activity may affect fertility even if PSA level or semen liquefaction appears to be normal. PSA is encoded by a gene that spans 12850 bp region on chromosome 19^24^. Genetic variations in *KLK3* gene could affect its activity and hence the degradation of semenogelins and the generation of active peptides, ultimately affecting fertility. In order to understand the contribution of *KLK3* genetic variations to infertility risk, we re-sequenced its complete coding region in 875 infertile and 290 fertile men. We identified a total of twenty-eight substitutions, of which five appear to be strong risk factors for male infertility.

## Materials and Methods

### Sample collection

We recruited 875 infertile men and 290 fertile controls from the King George’s Medical University (KGMU), Lucknow and the Institute of Reproductive Medicine (IRM), Kolkata. The study was approved by the Institutional Human Ethics Committee of the Central Drug Research Institute (CDRI), Lucknow. All experiments were performed in accordance with the relevant guidelines and regulations of the Institutional Ethics Committee. A verbal explanation of the nature of study was given to participants while taking their informed written consent.

The inclusion criteria for infertile patients was based on infertility persisting longer than one year and absence of any obvious fertility problem in the partner (menstruation and ovulation). A detailed clinical workout on the female partner was taken as the absence of any abnormality in her and to narrow down the problem to the male partner. Sperm count and motility in the case group were between 0 and 200 (average = 54.4) and between 0 and 85 (average = 7.4%), respectively. Male individuals exhibiting obstruction to sperm release, varicocele, endocrine imbalance, infection of accessory glands and human immunodeficiency virus positivity were excluded from the study. Semen analysis was performed after an abstinence of 3–7 days. The patients pool consisted of individuals with oligozoospermia (N = 68), azoospermia (N = 279), asthenozoospermia (N = 246) or normozoospermia (N = 149), uncategorized (N = 133), but experiencing infertility after at least one year of unprotected intercouse constituted the infertile group. The patients were identified from their visits to the clinic on their own or by referal. Most of the patients had been trying for parenthood for the last more than three years. The controls were recruited following the criteria of confirmed paternity. Semen samples for all control samples were not obtained, but confirmed paternity in the last two years was taken as a proof of their fertility. Sperm count and motility in the control group were between 35 and 180 (average = 89.1), and between 34 and 85 (average = 68.7%), respectively. The study subjects (cases and controls) were of Indo-European ethnicity with an average age of 34.13 ± 6.16 years. The average age was 33.11 for the case group and 35.15 for the control group.

### Genomic DNA Isolation and DNA sequencing

Genomic DNA was isolated from the peripheral blood samples of subjects using phenol-chloroform isoamyl method as described previously^25^. Sequence of the *KLK3* gene was retrieved from the Ensembl database (Gene ID: ENSG00000142515), and primers for the coding region were designed using the primer-blast tool available at NCBI. Primers were custom synthesised by Eurofins, Bangalore, India. PCR amplification was carried out as previously described^26^ with details provided in Table 1. Amplicons were treated with Exo-Sap (Exonuclease I and Shrimp Alkaline Phosphatase, ExoSAP-IT; USB Corporation, Cleveland, OH, USA) to remove unutilized primers and dNTPs as per the manufacturer’s protocol. Direct DNA sequencing using BigDye^TM^ chain termination chemistry was performed on ABI 3730 DNA analyzer (Applied Biosystems, USA)^27^. Multiple alignment and sequence analysis were done using Auto Assembler Software (Applied Biosystems, USA).

**Table 1:** Details of primers and PCR coditions used for the amplification of KLK3 exons.

Primer Pair	Primer sequences (Forward/Reverse)	Annealing temperature (°C)	Product size (bp)
1	GGGGGTTGTCCAGCCTCCAGCAG/GCGGGGACCTGGTGTGGGAGTG	63	404
2	GCCCCCGTGTCTTTTCAAACCC/TCCCATGCGTGTGCTCAGTAGG	65	708
3	TGCCCTTCACCCTCTCACACTG/GGGGTCAAGACTACGGGCCAGGC	63	509
4	GGTGCAGCCGGGAGCCCAGATG/CGGGGAGGTGGCATGGCTACAG	65	391
5	GGGGGTGGCTCCAGGCATTGTCC/AGGGGGTTGATAGGGGTGCTC	65	722
6	GGTGTGAGGTCCAGGGTTGCTAGG/CCACTGGGAGAAAACAACTGAAAG	65	634

### Total protein and t- PSA level in seminal plasma

Semen samples were centrifuged first at low speed (5000 rpm) for 10 minutes at 4 °C and later at high speed (12000 rpm) for 10 minutes at 4 °C for obtaining the seminal plasma from infertile men. Total protein content in the seminal plasma was assessed by Bradford method. Seminal plasma was diluted 1000 times for estimation of t-PSA using an ELISA based kit from Weldon Biotech (Cat No: t-PSA 118WB). Absorbance was measured on µQuant (Bio-Tek Instruments Inc.) and analyzed using KC Junior software.

### Statistical analysis

Chi square analysis was used to compare genotypes frequency between fertile and infertile men using the VassarStats Online Calculator (http://faculty.vassar.edu/lowry/VassarStats.html). Odds ratio (OR) was calculated using the dominant model of analysis for all the substitutions. Linkage disequilibrium between each pairwise combination of SNPs and haplotype frequencies were calculated using the Haploview software (Version 4.2) (http://www.broadinstitute.org)^28^. A P-value of less than 0.05 was considered to be statistically significant.

### In silico analysis

Variant effect prediction analysis was done using the VEP tool available at the Ensembl database (www.ensembl.org). PolyPhen (Polymorphism Phenotyping) (http://genetics.bwh.harvard.edu/pph2/) and SIFT (Sorting Intolerant from Tolerant) (http://siftdna.org/www/Extended_SIFT_chr_coords_submit.html) scores were used for the prediction of functional impact of the non-synonymous substitutions. PolyPhen and SIFT scores predict the effect of an amino acid substitution on structure and function of a protein, using sequence homology, proximity of the substitution to predicted functional domains or structural features, and physicochemical similarity between alternate amino acids.

### Data availability

All data generated or analysed during this study are included in this published article.

## Results

**Figure 1:**
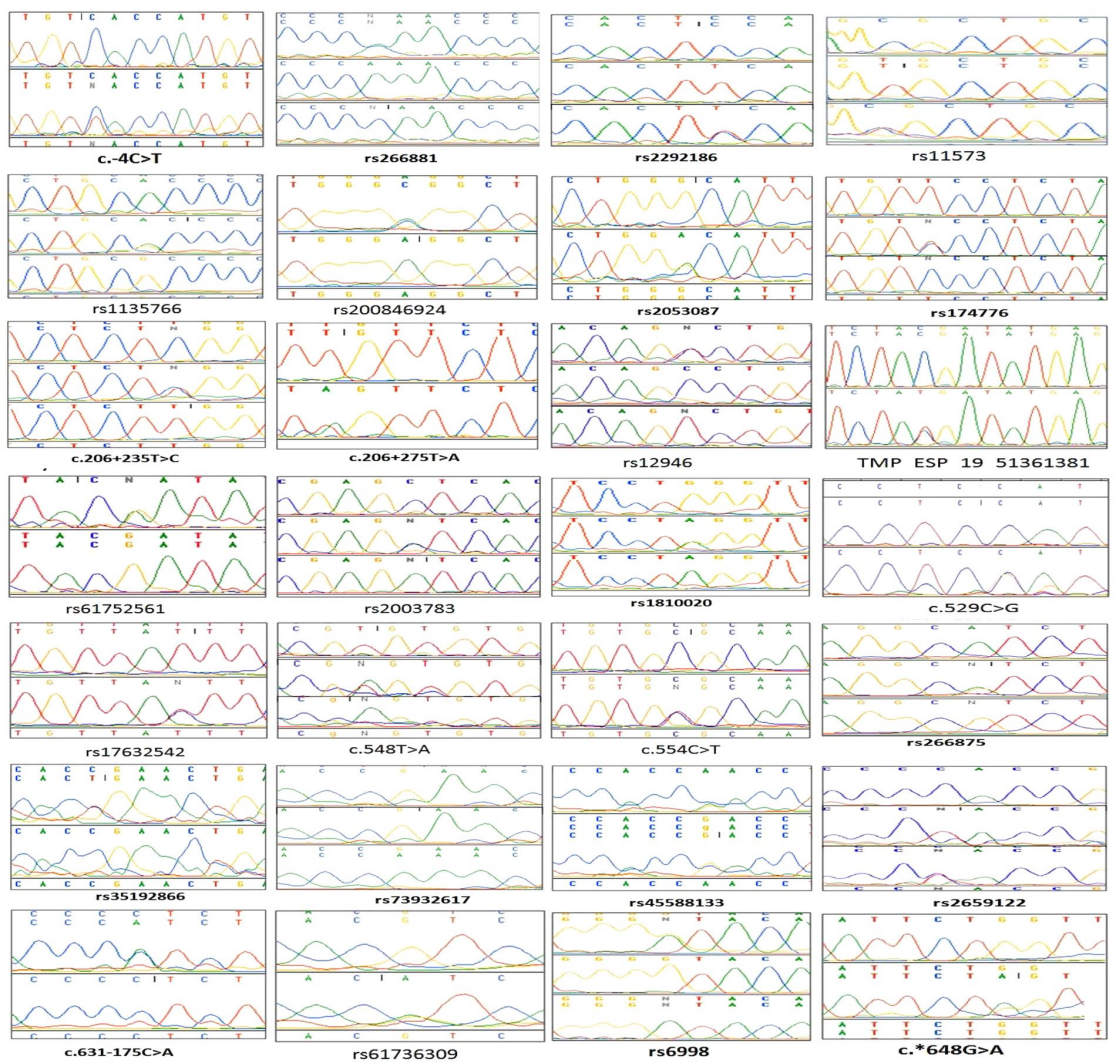
Electropherograms of the substitutions identified.

Sequence analysis identified twenty-eight substitutions (Figure 1 and Table 2), of which eight were called mutations (present in <1% frequency) and rest were labelled as SNPs (present in >1% frequency). Variant effect prediction revealed six to be missense variants, five to be synonymous variants, thirteen to be intronic variants, one to be a splice region variant, one to be a 5′UTR variant, one to be 3′UTR variants, and one to be a downstream gene variant with reference to the transcript **ENST00000326003**. Upon further investigation, two of the intronic substitutions were found to be missense with reference to other transcripts (**ENST00000597483** and **ENST00000596185**). Out of twenty-eight substitutions, eight were novel that had not been catalogued in the dbSNP or ESP databases. Nomenclature of the novel substitutions was done following the guidelines of the Human Genome Variation Society (HGVS) (http://www.hgvs.org/mutnomen/). The non-synonymous substitutions, c.529 C > G, c.548 T > A, c.554 C > T, rs61752561, rs2003783, rs17632542, rs266881, and rs73932617 resulted in p.His177Asp (**ENST00000326003**), p.Val183Glu (**ENST00000326003**), p.Ala185Val (**ENST00000326003**), p.Asp102Asn (**ENST00000326003**), p.Leu132Ile (**ENST00000326003)**, p.Ile179Thr (**ENST00000326003)**, p.Pro41Gln (**ENST00000596185**), and p.Glu174Lys (**ENST00000597483**) changes, respectively (Table 2). *In silico* analysis using Polyphen predicted none of these to be functionally ‘damaging’ and *in silico* analysis using SIFT predicted p.Glu174Lys, and p.Ile179Thr to be ‘deleterious’.

**Table 2:** Minor allele frequency distribution of KLK3 gene substitutions in infertile and fertile men.

S.No.	Substitution	Location as per GRCh37	rs number	Nomenclature	Amino acid Change	Polyphen prediction	SIFT prediction	Minor allele	Minor allele frequency (MAF)		Reported MAF
									Infertile	Fertile	
1	C/T	51358208	Novel	c.-4C > T				T	0.0041	0.004	
2	C/A	51358333	rs266881		P/Q^*^	0	—	A	0.4	0.22	0.38
3	C/T	51358394	rs2292186					T	0.16	0.18	0.25
4	T/C	51359497	rs11573					C	0.06	0.0	0.40
5	A/G	51359503	rs1135766					G	0.034	0.0	0.40
6	A/C	51359530	rs200846924					C	0.001	0.004	—
7	G/A	51359716	rs2053087					A	0.017	0.002	—
8	T/C	51359852	rs174776					T	0.485	0.615	0.21
9	T/C	51359890	Novel	c.206 + 235 T > C				C	0.04	0.09	
10	T/A	51359930	Novel	c.206 + 275 T > A				A	0.001	0.002	
11	C/T	51361315	rs12946					T	0.054	0.062	0.10
12	C/T	51361381	TMP_ESP_19_51361381					T	0.003	0.0	–
13	G/A	51361382	rs61752561		D/N	0.001	1	A	0.011	0.005	0.01
14	C/A	51361472	rs2003783		L/I	0.004	1	A	0.042	0.033	0.10
15	A/G	51361644	rs1810020					A	0.299	0.371	0.20
16	C/G	51361750	Novel	c.529 C > G	H/D	0.001	–	G	0.0024	0.0	
17	T/C	51361757	rs17632542		I/T	0.069	0.02	C	0.026	0.026	0.03
18	T/A	51361769	Novel	c.548 T > A	V/E	0.002	>0.05	A	0.00	0.061	
19	C/T	51361775	Novel	c.554 C > T	A/V	0.414	>0.05	T	0.0006	0.0	
20	G/A	51361937	rs266875					G	0.46	0.51	0.42
21	C/T	51362803	rs35192866					T	0.035	0.013	0.09
22	G/A	51362804	rs73932617		E/K^#^	0.116	0.0	A	0.027	0.0	0.03
23	G/A	51362955	rs45588133					A	0.04	0.045	0.09
24	C/T	51363026	rs2659122					T	0.46	0.36	0.40(C)
25	C/A	51363053	Novel	c.631–175 C > A				A	0.015	0.00	
26	G/A	51363278	rs61736309					A	0.004	0.00	<0.01
27	G/A	51363661	rs6998					A	0.196	0.152	0.31
28	G/A	51364030	Novel	c.*648 G > A				A	0.003	0.00	

*2 with ref to ENST00000596185; ^#^24 with ref to ENST00000597483; Rest according to ENST00000326003.

### Comparison based on fertility status

Minor allele frequencies for each variation are detailed in Table 2. Eight substitutions (rs11573, rs1135766, rs73932617, c.206 + 56 G > A, c.529 C > G, c.554 C > T, c.631–175 C > A, TMP_ESP_19_51361381, c.631–116 T > C, rs61736309, and c.*648 G > A) were observed exclusively in infertile men and one (c.548 T > A) exclusively in fertile men. Genotype distributions for six SNPs (rs266881, rs174776, c.206 + 235 T > C, rs1810020, rs266875 and rs35192866) were significantly different between fertile and infertile men according to 2 by 3 contingency (Table 3, dominant model). Five of these substitutions (rs266881, rs174776, rs1810020, rs266875, rs35192866,) increased the risk of infertility, while one (c.206 + 235 T > C) was protective. The frequency of ‘CA + AA’, ‘TC + CC’, ‘AA + AG’, ‘GG + GA’ and ‘CC + CT’ genotypes for SNPs rs266881 (OR = 2.88, P = <0.0001), rs174776 (OR = 1.91, P =<0.0001), rs1810020 (OR = 2.08, P = 0.034), rs266875 (OR = 1.44, P = 0.016) and rs35192866 (OR = 4.48, P = 0.025), respectively, were significantly higher in the infertile group as compared to the fertile group. On the contrary, the frequency of ‘TC + CC’ genotype for c.206 + 235 T > C (OR = 0.44, P = 0.002) was higher in fertile controls. At least four of these correlations were confirmed by 2 × 3 contingency table analysis as well.

**Table 3:** Statistical comparison of the genotype distribution of identified SNPs between infertile and fertile.

S.No.	rs number	Mutation	Infertile vs Fertile	
			2 × 3	11 vs (12 + 22)
1	c.−4C > T	C/T	0.99	1.20 (0.20–11.59); 1.0
2	rs266881	C/A	<0.0001^*^	2.88 (2.07–4.02); <0.0001^*^
3	rs2292186	C/T	0.164	1.58 (0.98–2.54); 0.057
4	rs11573	T/C	Observed in infertile group only	
5	rs1135766	A/G	Observed in infertile group only	
6	rs200846924	A/C	0.45	0.24 (0.022–2.68); 0.25
7	rs2053087	G/A	0.15	5.73 (0.74–44.29); 0.072
8	rs174776	T/C	<0.0001^*^	1.91 (1.47–2.49); <0.0001^*^
9	c.206 + 235 T > C	T/C	0.004^*^	0.44 (0.26–0.75); 0.002^*^
10	c.206 + 275 T > A	T/A	0.92	0.57 (0.04–9.09); 1.0
11	rs12946	C/T	0.29	0.92 (0.57–1.51); 0.75
12	TMP_ESP_19_51361381	C/T	Observed in infertile group only	
13	rs61752561	G/A	0.49	2.34 (0.52–10.45); 0.38
14	rs2003783	C/A	0.12	1.39 (0.75–2.55); 0.29
15	rs1810020	A/G	0.099	2.08 (1.05–4.13); 0.034^*^
16	c.529 C > G	C/G	Observed in infertile group only	
17	rs17632542	T/C	1.0	1.0 (0.54–1.85); 1.0
18	c.548 T > A	T/A	Observed in fertile group only	
19	c.554 C > T	C/T	Observed in infertile group only	
20	rs266875	G/A	0.028^*^	1.44 (1.07–1.93); 0.016^*^
21	rs35192866	C/T	0.084	4.48 (1.06–18.9); 0.025^*^
22	rs73932617	G/A	—	—
23	rs45588133	G/A	0.275	1.02 (0.43–2.39); 1.0
24	rs2659122	C/T	0.287	1.30 (0.94–1.80); 0.12
25	c.631–175 C > A	C/A	Observed in infertile group only	
26	rs61736309	G/A	Observed in infertile group only	
27	rs6998	G/A	0.49	1.19 (0.84–1.69); 0.32
28	c.^*^648 G > A	G/A	Observed in infertile group only	

*p < 0.05, was considered statistically significant.

### Linkage disequilibrium and haplotype analysis

**Figure 2:**
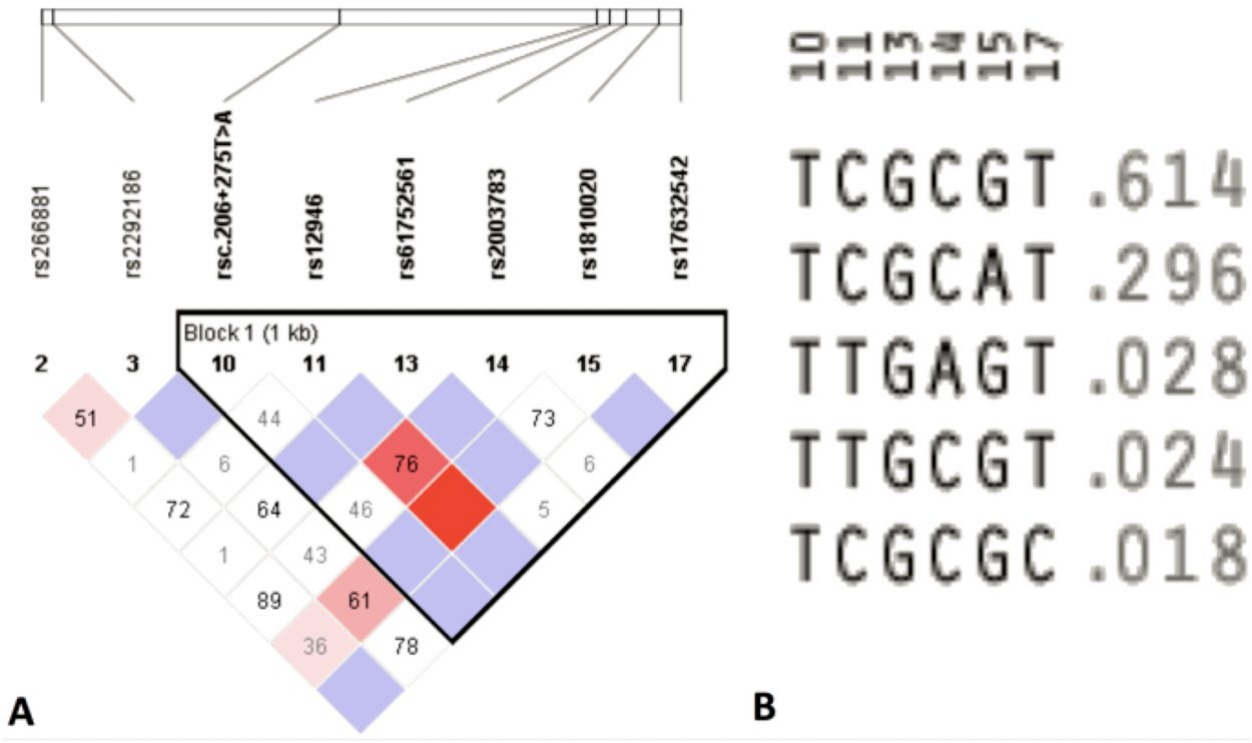
LD analysis using four gamete rule method showing LD (**A**) and haplotypes (**B**).

Out of 28 SNPs, eight SNPs (rs266881, rs2292186, rsc.206 + 275 T > A, rs12946, rs61752561, rs2003783, rs1810020, and rs17632542) qualified for the haploview analysis. Three SNPs (rsc.−4C > T, rs200846924, and rsc.554 C > T) had failed due to low maf (MAF <0.001). Five SNPs (rs2053087, rs174776, rsc.206 + 235 T > C, rsc.548 T > A, and rs266875) failed the HWE test (<0.001). Six SNPs (rs35192866, rs73932617, rs45588133, rsc.631–175 C > A, rs61736309, and rsc.*648 G > A) failed the % genotypes test (cut off value = 75%). Six SNPs (rs11573, rs1135766, rsTMP_ESP_19_51361381, rsc.529 C > G, rs2659122 and rs6998) failed both the HWE test and % genotype test. Therefore, LD analysis was performed on the reamining eight SNPs. We performed LD analysis using four gamete rule method, which created one block with six SNPs (rsc.206 + 275 T > A, rs12946, rs61752561, rs2003783, rs1810020, and rs17632542) showing strong LD (Fig. 2A). Haplotype analysis of this block revealed that TCGCGT haplotype had the highest frequency (f = 0.614) among other haplotypes (TCGCAT = 0.296; TTGAGT = 0.028; TTGCGT = 0.024; TCGCGC = 0.018) (Fig. 2B). Distribution of these haplotypes between cases and controls revealed no significant difference (Table 4).

**Table 4:** Haplotype analysis based on four gamete rule method.

Block	Haplotype	Freq.	Case, Control Ratio Counts	Case, Control Frequencies	Chi Square	P Value
Block 1						
	**TCGCGT**	0.614	506.2:325.8, 297.5:178.5	0.608, 0.625	0.347	**0.5561**
	**TCGCAT**	0.296	248.6:583.4, 139.0:337.0	0.299, 0.292	0.065	**0.7993**
	**TTGAGT**	0.028	19.7:812.3, 16.8:459.2	0.024, 0.035	1.489	**0.2223**
	**TTGCGT**	0.024	20.1:811.9, 11.4:464.6	0.024, 0.024	0.001	**0.9799**
	**TCGCGC**	0.018	15.5:816.5, 8.0:468.0	0.019, 0.017	0.054	**0.8161**

**Figure 3:**
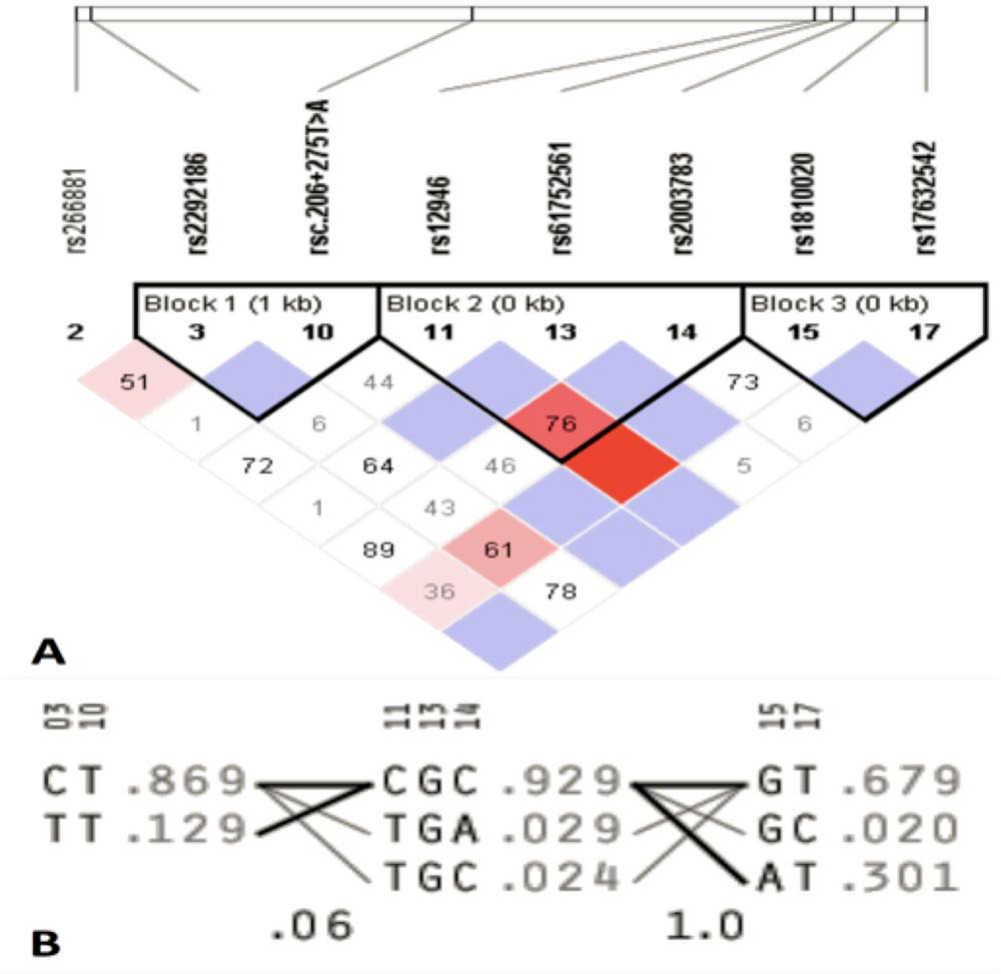
LD analysis by solid spine of LD method showing LD (**A**) and haplotypes (**B**).

LD analysis by solid spine of LD method created three blocks. Block 1 contained 2 SNPs (rs2292186, rsc.206 + 275 T > A), block 2 contained three SNPs (rs12946, rs61752561, rs2003783,) and block 3 contained 2 SNPs (rs1810020, and rs17632542) (Fig. 3A). Haplotype analysis of these blocks revealed that block 1, 2 and 3 had the highest frequency of haplotypes CT (f = 0.869), CGC (f = 0.929), and GT (f = 0.679), respectively (Fig. 3B). Further, the distribution of haplotypes between cases and controls revealed no significant difference (Table 5).

**Table 5:** Haplotype analysis based on solid spine of LD method.

**Haplotype**	**Freq**.	**Case, Control Ratio (Counts)**	**Case,Control Frequencies**	**Chi Square**	**P Value**
**Block 1**					
CT	0.869	660.1: 109.9, 421.2: 52.8	0.857, 0.889	2.533	**0.1115**
TT	0.129	108.6: 661.4, 51.7: 422.3	0.141, 0.109	2.684	**0.1014**
**Block 2**					
CGC	0.929	757.1: 60.9, 444.6: 31.4	0.926, 0.934	0.326	**0.5678**
TGA	0.029	21.2: 796.8, 16.9: 459.1	0.026, 0.035	0.964	**0.3263**
TGC	0.024	19.9: 798.1, 11.4: 464.6	0.024, 0.024	0.002	**0.9631**
**Block 3**					
GT	0.679	569.3: 272.7, 344.8: 159.2	0.676, 0.684	0.093	**0.761**
AT	0.301	254.6: 587.4, 150.2: 353.8	0.302, 0.298	0.028	**0.8666**
GC	0.02	18.1: 823.9, 9.0: 495.0	0.022, 0.018	0.214	**0.6439**

### Estimation of t-PSA in seminal plasma

We quantified total PSA in seminal plasma of 96 infertile men and correlated its concentration with semen liquefaction time and sperm motility. We did not find any significant correlation of t-PSA level in seminal plasma with either motility (0.085) or liquefaction time (−0.062).

## Discussion

Since long, PSA is well known to be the chief executor of the process of semen liquefaction, which releases the mass of entangled spermatozoa to achieve active motility and initiate their journey towards the ovum. Complete or partial failure of semen liquefaction would result in the loss of sperm motility, causing or contributing to infertility. Men with reduced sperm motility had low seminal fluid PSA^29, 30^ and a study on Swedish men showed a direct association between PSA level in the seminal fluid and sperm motility in normal male population^23^. In a large number of infertility cases, PSA is produced in sufficient quantity and semen liquefaction takes place within 5–20 minutes; this may exclude PSA as a possible cause of infertility in these cases. However, studies in the last two decades have pointed out that PSA may have a long trail of its impact of sperm functions and fertility that go beyond semen liquefaction. This starts in the vagina (site of semen deposition) with the first step in the form of semenogelins fragmentation, releasing sperm. Hereafter, semenogelins and their fragments are thought to affect sperm fertility by increasing thyrotropin releasing hormone like action, promoting zinc shuttling and inhibin like activities^22^.

The whole of seminal plasma contents are left behind once sperm make their way into the uterus. However, semenogelin like peptide fragments have been reported in sperm fractions in a number of studies^2, 17, 31–33^. A 19 kDa protein (probably from semenogelin processing) was found at the periphery of detergent-treated human sperm nuclei^31^. Further, the high binding capacity of semenogelins and their fragments for Zn^2+^ promotes the shuttling of Zn^2+^ to sperm nucleus, where it plays essential role in DNA stability^2, 17^. An interesting study found a 21 kDa protein (identified as semenogelins I precursor) in spermatozoa that was found at higher concentration in asthenozoospermic infertile men^33^. Among other evidences in support of numerous functions of semenogelins in sperm fertility, a recent study provided unequivocal evidence that semenogelins in fact cross the sperm plasma membrane to serve intracellular functions such as the inhibition of capacitation^10^. The study also reported that the levels of semenogelins drop fast at the time of sperm capacitation with a rise in ROS generation.

The above functions of semenogelins are dependent on PSA and other KLKs, making them important for fertility. Erroneous processing of semenogelins could have impact on sperm motlity/fertility even if semen liquefaction appears normal. It is possible that higher level of sperm semenogelins in some infertile men^33^ and its slow degradation^32^ could delay or prevent capacitation. Therefore, optimal activity of PSA and other KLKs is critical for sperm fertility. Among studies on KLK genes, Lee and Lee (2011) genotyped *KLK2* SNPs (+255 G > A, rs2664155) in 218 infertility cases and 220 fertile controls and found a significant correlation of the polymorphisms with male infertility^34^. Savblom *et al*.^35^ reported the association of few SNPs in the h*KLK2* and *PSA* genes with seminal and serum levels of KLK2 and PSA levels^35^. Similarly, a previous study reported a strong association of *KLK7* polymorphisms with semen hyperviscosity, with a higher incidence in infertile cases^36^.

We identified 28 substitutions, out of which five (rs266881, rs174776, rs1810020, rs266875, rs35192866) associated with increased risk of infertility, while one (c.206 + 235 T > C) was protective. LD analysis suggested three blocks of SNPs, but haplotype analysis revealed no significant difference between cases and controls. SNPs (rs266881, rs174776) that fall in the intronic region of transcript **ENST00000326003** may affect regulatory functions by as yet unknown mechanisms. Substitution at rs266881 results in a non-synonymous change in the transcript **ENST00000596185** and increases the risk of infertility. This is the first study reporting the association of *KLK3* SNPs with male infertility; however, the functional significance of these polymorphisms remains to be worked out. The genetic variations in kallikreins in relation to their impact on male fertility is in infantile stage and further studies on other candidate kallikreins are required. We did not find a correlation between PSA concentration and semen liquefaction/sperm motility; nevetheless, a previous study reported a significant correlation between PSA level and sperm motility in normal Swedish men^23^ and another study reported a similar correlation in infertile individuals^37^. This suggests that PSA level could affect sperm motility, but the effect of PSA activity on sperm motility and fertility has not been assessed.

In a nutshell, the increasing understanding of the functions of semenogelins and their petides in sperm fertility makes PSA far more than imporant for male fertility than previously thought. Eight of twenty-eight substitutions we observed had not been reported in the dbSNP and ESP databases before. Out of twenty-eight, only five SNPs correlated with increased infertility risk in our population; however, studies on other populations would help in identification of the most common risk factors for male infertility. SNPs rs266881, rs174776, rs1810020, rs266875 and rs35192866 affect the risk of male infertility and merit further investigation in other populations. Nevertheless, there were other SNPs which were observed solely in infertile cases, but their absence in controls may be a chance event. Therefore, KLK3 analysis in infertile individuals from ethnically different populations is strongly recommended. The findings of the present study would open up new horizons for investigation of KLK3′s importance in male fertility. Kallikrein related peptidases are so important in fertility that a host of them are found in cervical-vaginal fluid as well^38^. Further studies on semenogelins and PSA may reveal far unanticipated roles that they play in sperm functions and male fertility.
